# Specific inhibition of p38α MAPK dampens neuroinflammation during acute alcohol withdrawal in mouse BV2 microglial cell line and rat organotypic hippocampal slice cultures

**DOI:** 10.1016/j.alcohol.2026.03.007

**Published:** 2026-03-27

**Authors:** M.C. Green, D.J. Braun, C.T. Leibold, L.J. Van Eldik, C.S. Bailey

**Affiliations:** a Sanders-Brown Center on Aging, University of Kentucky College of Medicine, USA; b Department of Neuroscience, University of Kentucky College of Medicine, USA; c Department of Psychology, University of Kentucky College of Arts & Sciences, USA

**Keywords:** BV2, Organotypic hippocampal slice culture, Alcohol withdrawal, Neuroinflammation, CXCL1, TNFα, p38α MAPK, MW150

## Abstract

Neuroinflammation is implicated in anxiety and negative affect in alcohol withdrawal, potentially contributing to relapse. The mitogen-activated protein kinase p38α (p38) is a critical driver of neuroinflammation in such excitatory neural contexts, and its inhibition reduces neuroinflammatory cytokine production in the context of various insults generally corresponding with improved cellular and synaptic health. Although heretofore unexamined, we hypothesized that inhibition of p38 by small-molecule MW150 would reduce neuroinflammation during the acute alcohol withdrawal period. Immortalized mouse BV2 and post-natal day 8 rat organotypic hippocampal slice cultures received 50mM ethanol in media for 24 h followed by 24 h withdrawal, or for 48 h continuously, with administration of 5 μM MW150 or saline for the final 24 h of treatment. Control tissue never received ethanol. Levels of cytokines in the culture media were analyzed after 48 h by MesoScale ELISA assays. Elevated CXCL1 and TNFα levels were ameliorated by MW150 during ethanol withdrawal in culture media from BV2 and female OHSC, respectively. Further, MW150 reduced TNFα, but increased IL6, across all conditions in the BV2 microglia. Preliminary evidence suggests that p38 inhibition during early ethanol withdrawal in vitro reduces select inflammatory cytokines. Given that MW150 is presently in clinical trials for neuroinflammation in Alzheimer’s disease, its preclinical validation for use in alcohol withdrawal in vivo is crucial to determine its feasibility to modulate neuroinflammation and problem drinking in humans.

## 1. Introduction

Alcohol use disorder (AUD) is a common and costly public health concern. It is characterized by a cyclical pattern of binge/intoxication, withdrawal/negative affect, preoccupation/anticipation, and chronic relapse (Koob & Volkow, 2016; Sliedrecht et al., 2019). In the brain, alcohol exposure increases production of pro-inflammatory chemokines/cytokines, such as interleukin 1β (IL-1β) (Baker et al., 2023; Qin et al., 2008), interleukin 6 (IL-6) (Gruol et al., 2023; Zago et al., 2016), tumor necrosis factor α (TNFα) (Kishore et al., 2001; Pascual et al., 2015), and C-X-C motif chemokine ligand 1 (CXCL1) (Baker et al., 2023). This enhanced neuroinflammation can induce widespread cellular damage and compound the negative affect and craving precipitated by withdrawal (Lékó et al., 2023), contributing to relapse and perpetuating the cycle of addiction (Koob, 2013).

Of the neuroinflammatory mosaic, TNFα is specifically related to increased cell death, excitatory neurotransmission, and anxiety (Alvarez Cooper et al., 2020) associated with alcohol exposure and subsequent withdrawal – as well as peripheral damage including alcoholic liver disease (ALD) (McClain et al., 1998). While TNFα inhibitors (e.g., infliximab and etanercept) ameliorate symptoms of alcohol-induced liver damage, such anti-TNFα therapies increase risk of infection and mortality in individuals with alcoholic hepatitis (Boetticher et al., 2008; Naveau et al., 2004). However, a critical modulator of TNFα release is the mitogen-activated protein kinase (MAPK) p38α (p38). P38 is an oxidative stress sensor that broadly orchestrates neuroinflammatory responses (Canovas & Nebreda, 2021) involved in various causes of brain injury (Asih et al., 2020), including those associated with alcohol use and withdrawal (Jung et al., 2011). Alcohol exposure can increase p38 activation (Norkina et al., 2007) which corresponds with sustained production of TNFα and other pro-inflammatory chemokines/cytokines (Falcicchia et al., 2020). Interestingly, inhibition of p38 in the presence of alcohol corresponds with a reduction in TNFα production in the rat liver (Kishore et al., 2004) and mitigated cell death in mouse hippocampal HT-22 cells (Ku et al., 2007).

Notably, these earlier studies relied on the p38 inhibitor SB203580 which (1) does not distinguish between the alpha- and beta-isoforms of p38, and (2) triggers off-target ERK signaling alterations at higher doses. More recently developed brain-penetrant p38 inhibitors that are specific to the alpha isoform (e.g., Neflamapimod and MW150) are currently in clinical trials for patients with dementia (Prins et al., 2021; Roy et al., 2015, 2019) based on a wealth of preclinical data. Of particular relevance to ethanol-induced neuropathology, p38α inhibition reduces neuroinflammation and tau dysregulation, preserves neuronal integrity, normalizes hippocampal electrophysiological dysfunction, and rescues behavioral alterations (Frazier et al., 2024; Maphis et al., 2016; Roy et al., 2019; Rutigliano et al., 2018). Taken together, p38 may be a suitable target for modulating neuroinflammation and associated damage during alcohol exposure and withdrawal in order to reduce the deleterious central and peripheral effects of repeated patterns of alcohol misuse.

We therefore evaluated the feasibility of the small-molecule p38α inhibitor MW150 (Roy et al., 2015, 2019) to reduce neuroinflammation during acute alcohol exposure and withdrawal using mouse microglial-type BV2 cells and neonatal rat organotypic hippocampal slice cultures (OHSC). We focused on TNFα, CXCL1, IL-1β, and IL-6 as proteins that are both controlled by p38α and implicated in ethanol-induced neuropathology. We found differential effects of p38α inhibition by MW150 across both BV2 and OHSC cultures during 48-h continuous ethanol exposure (CE) and 24-h ethanol withdrawal after 24 h of exposure (EWD). Importantly, we did not detect any additive toxicity of MW150 and ethanol in either model, consistent with other studies indicating protective effects of p38 suppression in ethanol exposure. In BV2 microglial cultures, TNFα was unaffected by ethanol but suppressed by MW150. IL-6 was increased above control levels by CE and EWD, and further increased by MW150. CXCL1 was elevated above control levels by both CE and EWD, but reduced by MW150 only in the latter. In the heterogeneous OHSC cell types, TNFα was elevated in EWD above control but reduced by MW150 treatment. CXCL1 levels were unaffected by either ethanol or MW150, and IL-6 levels were undetectable. In this report, we demonstrate the potential of MW150 to ameliorate the neuroinflammatory changes caused by alcohol withdrawal. Further assessment of such effects *in vivo* is warranted given the potential for drug repurposing.

## 2. Materials and methods

### 2.1. Mouse BV2 microglial cell culture

BV2 cells were grown and passaged as previously published (Bachstetter et al., 2011; Zhou et al., 2017). Cell growth media consisted of DMEM/F12 (Mediatech #15-090-CV; Manassas, VA) supplemented with 10% fetal bovine serum (HyClone #SH30071.03; Logan, UT), 1% penicillin-streptomycin (Mediatech #30-002-CI), and 1% L-glutamine (Mediatech #25-005-CI), and cells were maintained in an incubator at 37 °C with 5% CO_2_. Confluent cells had growth media removed and were detached from the T25 growth flask (Corning Falcon #353108; Corning, NY) with 0.05% Trypsin/0.53 mM EDTA in Hanks’ Balanced Salt Solution (HBSS; Mediatech #25-052-CI) for 2 min. Growth media was added to neutralize the trypsin, cells were pelleted at 500×*g*, and resuspended in fresh growth media. Cells were plated into 48-well plates at a final density of 100,000 cells/mL and volume of 200 μL per well (20, 000 cells/well). Cells were returned to the incubator overnight prior to treatment.

### 2.2. BV2 experimental design

Treatment media consisted of the same components as the growth media, but without fetal bovine serum. On day 1 of treatment, growth media was removed and replaced with 200 μL of treatment media or treatment media with 50 mM ethanol. Twenty-four hours later, media was removed and replaced with treatment media with saline, (2) treatment media with 50 mM ethanol, (3) treatment media with 5 μM MW150, or (4) treatment media with 50 mM ethanol and 5 μM MW150. This created 6 total conditions, each with 8 replicates: (1) no ethanol saline, (2) no ethanol MW150, (3) continuous ethanol saline, (4) continuous ethanol MW150, (5) ethanol withdrawal saline, and (6) ethanol withdrawal MW150. Cells were incubated for an additional 24 h.

### 2.3. BV2 sample preparation

Twenty-four hours after the treatment on day 2, media was removed and spun for 10 min at 1000×*g* prior to neuroinflammation analysis. Cells in the plate were subsequently washed in ice cold PBS, and 75 μL of MSD Tris lysis buffer (Mesoscale Discovery #R60TX; Rockville, MD) added per well. Plates were subsequently shaken at 4 °C for 5 min at 1000 RPM, homogenates spun down at 12,000×*g*, and supernatants frozen at −80°C prior to the BCA assay.

### 2.4. BV2 BCA and MesoScale discovery ELISA

BCA assay (ThermoFisher #23225; Waltham, MA) was performed according to manufacturer’s instructions without dilution. Neuroinflammation analysis was performed by analyzing 50 μL of culture media per well with a custom MesoScale Discovery VPLEX Proinflammatory Panel 1 Kit (#K15084D) according to manufacturer’s instructions for convergent analytes between p38 activity and alcohol exposure: IL-1β, IL-6, CXCL1, and TNFα (Bussi et al., 2017; Nick et al., 2000). Chemokine/cytokine values were normalized to the total BV2 protein level in each well. IL-1β was not analyzed further due to >20% of values undetected.

### 2.5. Rat organotypic hippocampal slice culture (OHSC)

This experiment was performed at the University of Kentucky in accordance with University of Kentucky IACUC protocols. Male and female Sprague Dawley rat pups (Inotiv Laboratories; Indianapolis, IN) were humanely euthanized at postnatal day 8 and tissue was harvested using aseptic technique as published (Bailey et al., 2022; Prendergast et al., 2000). Rat brains were removed and placed into cold dissecting media: 97.09% (v/v) Minimum Essential Media (MEM; Invitrogen; Carlsbad, CA), 0.024 M 4-(2-Hydroxyethyl)-1-piperazineethanesulfonic acid (HEPES; Sigma; St. Louis, MO), 0.97% penicillin/streptomycin (Invitrogen), and 1.94% Amphotericin B solution (Sigma). Hippocampi were extracted from both hemispheres and sectioned into 200 μm thick slices using the McIllwain Tissue Chopper (Mickle Laboratory Engineering Co. Ltd.; Gomshall, UK). Sectioned tissue was then placed into petri dishes filled with culture media: 49.26% dissecting media (detailed above), 22.17% double distilled water, 24.63% Heat Inactivated Horse Serum (HIHS; Sigma), 2.46% HBSS (Invitrogen), 0.49% penicillin/streptomycin (Invitrogen), and 0.99% Amphotericin B solution (Sigma). From there, slices containing the CA1, CA3 and dentate gyrus (DG) hippocampal subregions were selected and plated onto Millicell biopore membrane inserts (0.4 μm, 30 mm diameter; Millipore; Burlington, MA), three hippocampal slices per insert. Inserts were placed into six-well culture plates (5 mL, 9.5 cm^2^ area; VWR; Radnor, PA) containing 0.5 mL of 37 °C culture media per well and placed into the incubator (37 °C at 5% CO_2_) for an initial five-day recovery period before random assignment to a treatment group. For the present study, 0.5 mL of culture media was sufficient for the initial five-day tissue recovery period and did not oversaturate the membrane, which has jeopardized tissue integrity in recent pilot experiments. After the initial five-day period at a 0.5 mL volume, culture media was replenished at a volume of 1 mL per well.

### 2.6. OHSC experimental design

Tissue was randomly assigned to one of six experimental groups, consistent with the BV2 experiment: Control (CTRL), CTRL + MW150, 48 h continuous ethanol exposure (CE), CE + MW150, 24 h ethanol withdrawal (EWD), and EWD + MW150. At 5 days *in vitro* (DIV), 50 mM of ethanol was administered in the culture media for the following groups: CE, CE + MW150, EWD, EWD + MW150. Twenty-four hours later at 6DIV, 50 mM of ethanol was administered to the CE and CE + MW150 groups. Additionally, 5 μM MW150 was added to the CTRL + MW150, EWD + MW150, and CE + MW150 groups, and unadulterated culture media was provided to the CTRL and EWD groups. Twenty-four hours later at 7DIV, media was collected for biochemical assays.

### 2.7. OHSC lactate dehydrogenase and MesoScale discovery ELISA assays

Lactate dehydrogenase (LDH) assay (Abcam #ab102526; Cambridge, UK) was performed on OHSC culture media at a 1:5 dilution. Formazan probe was read at 450 nm at 10 min and 30 min during 37 °C incubation. The difference in absorption between 30 min and 10 min was made the singular value of relative NADH concentrations among groups. Cytokine assay, as above, was performed on 50 μL of culture media per well with the custom MesoScale Discovery VPLEX Proinflammatory Panel 1 Kit (#K15084D) for IL-1β, IL-6, CXCL1, and TNFα, according to manufacturer’s instructions. IL-1β and IL-6 were not analyzed further due to >20% of values undetected.

### 2.8. Statistical analyses

Statistical analyses were performed using GraphPad Prism 10.3.1 (San Diego, CA). Each statistical test is included in the results section under the respective endpoint.

## 3. Results

The purpose of these experiments was to determine the potential utility of the brain-penetrant small molecule p38 inhibitor, MW150, in the context of ethanol exposure and withdrawal. Specifically, we found that MW150 altered cytokines implicated in ethanol-induced neural damage across different ethanol regimens in both BV2 and OHSC experiments.

### 3.1. Mouse BV2 microglial culture

We used mouse BV2 cultures to first determine if MW150 could modulate immune response in microglial-type cells in the context of continuous ethanol (48 h) exposure and/or ethanol withdrawal (24 h exposure, 24 h withdrawal). Cell culture supernatants were collected and assayed for cytokine levels as described above.

#### 3.1.1. Cytokine assay for CE and EWD in BV2 cultures

##### CXCL1.

**Fig. 1.**
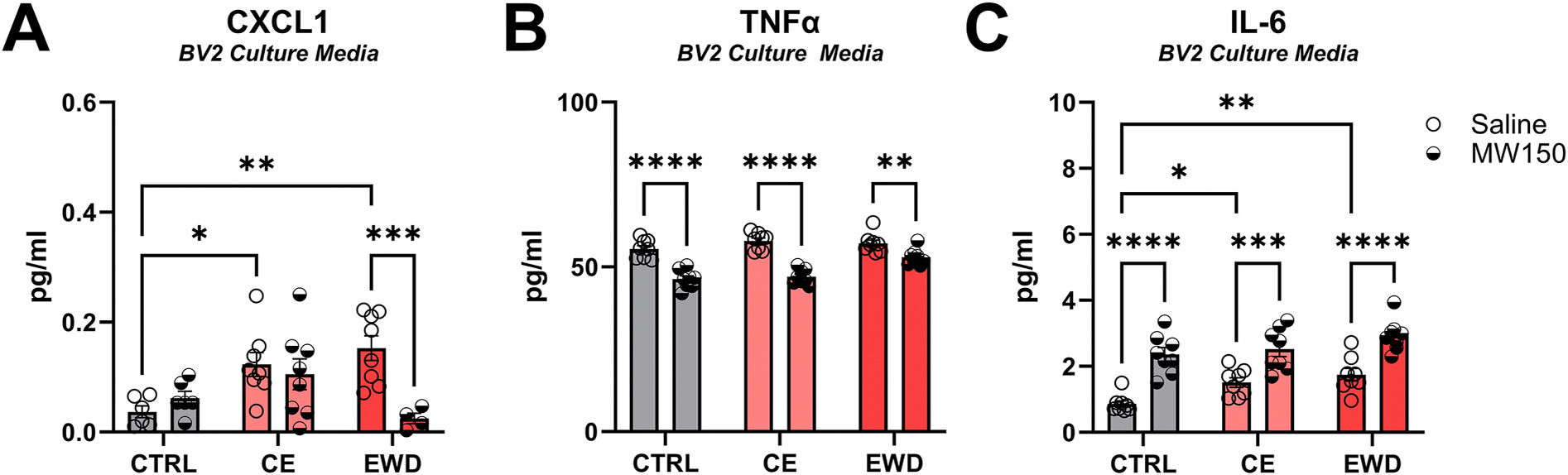
Chemokine/cytokine assay in BV2 culture media. **A**) In Saline-treated cells, both the CE and EWD regimens increased CXCL1 levels relative to Saline-treated CTRL cells. In the EWD group, MW150 reduced CXCL1 concentrations relative to Saline treatment. n = 40. **B**) MW150 reduced TNFα across all treatment conditions. n = 48. **C**) In Saline-treated cells, both the CE and EWD regimens increased IL-6 levels relative to Saline-treated CTRL cells. Further, regardless of condition, MW150 increased IL-6 levels relative to Saline. n = 48. *p* < *0.05, **0.01, ***0.001, ****0.0001. CTRL = control; CE = continuous ethanol; EWD = ethanol withdrawal.

There was an interaction between ethanol regimen and MW150 administration, *F* (2, 34) = 5.499, *p* < 0.01. Both CE (*M* = 0.12, *SD* = 0.06) and EWD (*M* = 0.15, *SD* = 0.06) regimens increased CXCL1 in Saline-treated cells relative to Saline-treated CTRL (*M* = 0.37, *SD* = 0.03) cells, *p* < 0.05 and *p* < 0.01, respectively. MW150 significantly reduced CXCL1 in the microglia of the EWD (*M* = 0.03, *SD* = 0.02) group compared to Saline in the EWD group, *p* < 0.001 (Fig. 1A). Two-way ANOVA with Tukey’s multiple comparison test.

##### TNFα.

There was an interaction between ethanol regimen and MW150 administration, *F* (2, 42) = 6.927, *p* < 0.01. MW150 treatment significantly reduced TNFα across CTRL (*M* = 46.33, *SD* = 2.80), *p* < 0.0001, CE (*M* = 47.04, *SD* = 2.22), *p* < 0.0001, and EWD (*M* = 52.98, *SD* = 2.46), *p* < 0.01, relative to the Saline treatment across CTRL (*M* = 55.47, *SD* = 2.81), CE (*M* = 57.85, *SD* = 2.55), and EWD (*M* = 57.15, *SD* = 2.87), *F* (2, 42) = 6.927, *p* < 0.01 (Fig. 1B). For BV2 treated with MW150, TNFα was higher in EWD relative to CE, *p* < 0.001, and CTRL, *p* < 0.0001. Two-way ANOVA with Tukey’s multiple comparisons test.

##### IL-6.

Any acute ethanol exposure increased IL-6 regardless of MW150 treatment, *F* (2, 42) = 8.53, *p* < 0.001. Further MW150 treatment produced more IL-6 compared to saline regardless of ethanol regimen, *F* (1, 42) = 71.68, *p* < 0.0001 (Fig. 1C). Two-way ANOVA.

#### 3.1.2. Effects of ethanol and MW150 on total protein in BV2 cultures

**Fig. 2.**
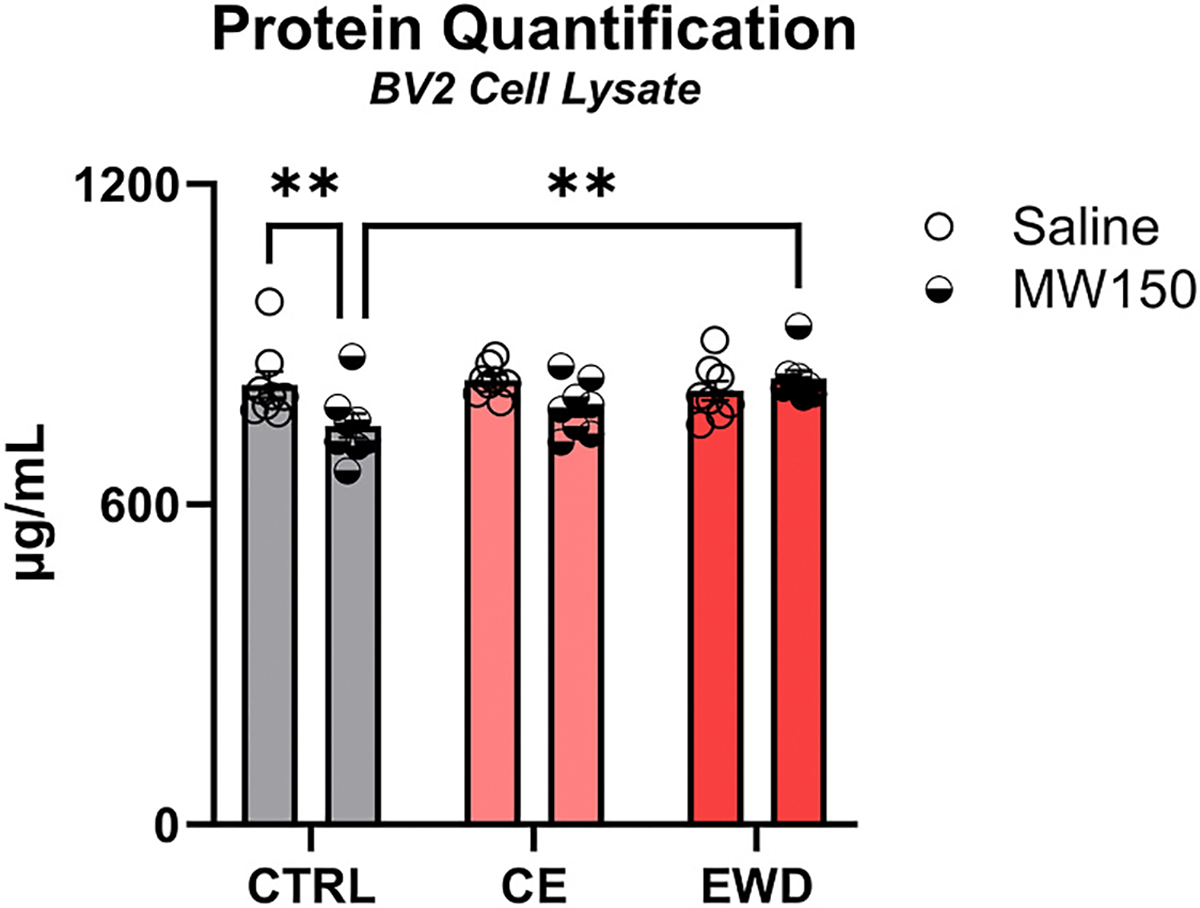
Assessment of potential interactive toxic effects of MW150 and ethanol in BV2 culture. Total protein was measured in cell lysates as an indicator of cell death. A slight decrease in protein was detected in cells treated with MW150 alone versus Saline control, but no additive decrement in protein levels was observed with ethanol and MW150 given together in the continuous nor withdrawal context. n = 48. *p* < **0.01. CTRL = control; CE = continuous ethanol; EWD = ethanol withdrawal.

There was an interaction between ethanol administration and MW150 treatment on total protein, *F* (2, 42) = 3.95, *p* < 0.05. Only in the CTRL condition, MW150 administration in the latter 24 h of the 48 h timeline reduced protein levels by 76.98 μg/mL relative to saline administration, *p* < 0.01 (Fig. 2). This effect is in line with other reports relating reduced proliferation with suppressed p38 action (Tikka et al., 2001; Wen et al., 2019). Interestingly, this effect was not detectable in either the CE or the EWD conditions. Further, protein levels were 88.82 μg/mL lower in the MW150 CTRL condition relative to the MW150 EWD condition, *p* < 0.01.

### 3.2. Rat OHSC

The BV2 experiment provides important proof-of-principle for the utility of MW150 in addressing ethanol-induced cytokine responses but is limited by the use of a single immortalized cell type. To better approximate the biological complexity, we next replicated the BV2 experimental design in rat OHSC tissue to evaluate the overall anti-inflammatory effects of MW150 in ethanol exposure and withdrawal in a condition closer to the *in vivo* context. There was no effect of ethanol administration nor MW150 treatment on TNFα or CXCL1 in OHSC culture media derived from male rat tissue. Therefore, we report only the effects observed in OHSC culture media derived from female rats.

#### 3.2.1. Cytokine assay for CE and EWD in OHSC media from female tissue

##### TNFα.

**Fig. 3.**
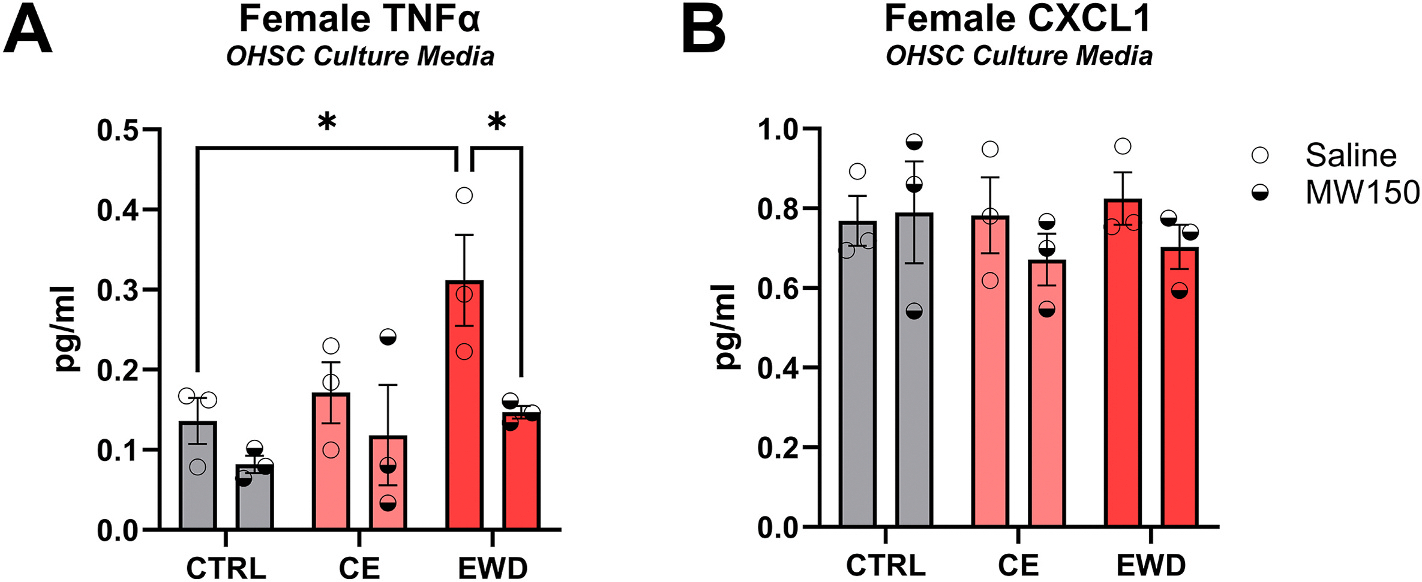
Chemokine/cytokine assay in OHSC culture media derived from female rats. **A**) In Saline-treated slices, EWD increased TNFα levels relative to CTRL slices. However, MW150 treatment decreased TNFα levels in EWD relative to Saline treatment. n = 18. **B**) There were no differences in CXCL1 across all conditions. n = 18. *p* < *0.05. CTRL = control; CE = continuous ethanol; EWD = ethanol withdrawal.

Ethanol regimen, *F* (2, 12) = 4.754, and MW150 treatment, *F* (1, 12) = 7.649 significantly affected TNFα in female OHSC tissue, *p* < 0.05. Specifically, in the Saline treatment group, EWD (*M* = 0.31, *SD* = 0.10) significantly increased TNFα levels relative to CTRL (*M* = 0.14, *SD* = 0.05), *p* < 0.05. Further, in the EWD condition, MW150 treatment (*M* = 0.15, *SD* = 0.01) significantly reduced TNFα levels relative to Saline (*M* = 0.31, *SD* = 0.10), *p* < 0.05 (Fig. 3A). Two-way ANOVA with Tukey’s multiple comparisons test.

##### CXCL1.

There were no significant differences in CXCL1 levels regardless of ethanol administration or MW150 treatment, *F* (2, 12) = 0.21, n.s. and *F* (1, 12) = 1.09, n.s., respectively (Fig. 3B).

#### 3.2.2. Lactate dehydrogenase (LDH) activity across conditions in OHSC

**Fig. 4.**
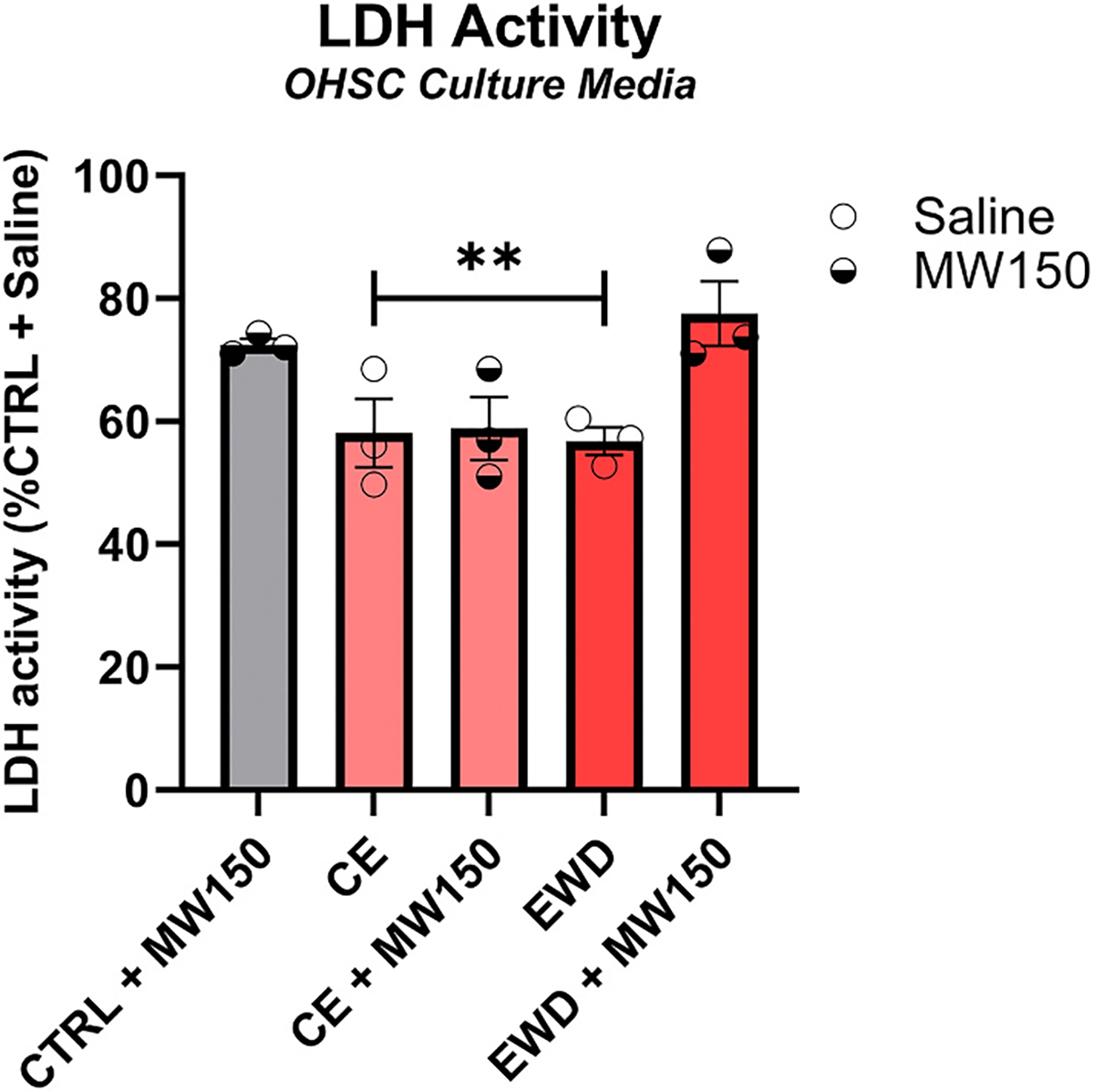
Assessment of potential interactive toxic effects of MW150 and ethanol in OHSC. LDH activity in culture media was measured as an indicator of cell death. LDH activity was not elevated above control levels in any of the conditions, consistent with no enhancement of cell death. n = 18. *p* < **0.01. CTRL = control; CE = continuous ethanol; EWD = ethanol withdrawal.

We measured LDH activity in OHSC media as a crude indicator of toxicity from concomitant ethanol and MW150 exposure. LDH activity in media was at or below CTRL levels across all conditions, in line with the lack of obvious cytotoxic effects in BV2 cells. Relative to CTRL, LDH was decreased in the CE ( ± MW150) and EWD groups, *p* < 0.01 (Fig. 4). One-way ordinary ANOVA with Tukey’s multiple comparisons test.

## 4. Discussion

In this study, we sought to determine the biological plausibility of attenuating the production of pro-inflammatory chemokines/cytokines via specific p38α inhibition during alcohol exposure and withdrawal using mouse BV2 microglial and rat OHSC models. In the BV2 cells, both CXCL1 and IL-6 were upregulated in the CE and EWD conditions, but p38α suppression by MW150 reduced CXCL1 in only the EWD and not the CE context. IL-6 was increased by MW150 regardless of context. TNFα was not elevated by either CE or EWD in the BV2 cells, but MW150 decreased the TNFα levels across all conditions. The only ethanol-responsive cytokine in our OHSC paradigm was TNFα, which was increased with EWD. Administration of MW150 was able to abolish this upregulation. While early and thus far limited to *in vitro* systems, these data provide initial proof-of-principle that the specific brain penetrant p38α inhibitors already in advanced clinical development may be useful in treating AUD. Our findings are explored below.

In this study, specific p38α inhibition by MW150 reduced elevated CXCL1 and TNFα in the context of alcohol withdrawal. These two inflammatory proteins are significantly implicated in central neuroinflammatory response to and peripheral liver damage arising from alcohol exposure – positioning them as crucial targets in comprehensively mitigating the effects of chronic alcohol misuse.

Specifically during alcohol withdrawal, the compensatory glutamate surge in the central nervous system (CNS) acts as a danger-associated molecular pattern (DAMP) which can activate toll-like receptors that, in turn, initiate neuroimmune responses via the p38 pathway. P38 then upregulates production of proinflammatory chemokines/cytokines, such as CXCL1 and TNFα (Lee et al., 2000; Lo et al., 2014). Both of these inflammatory mediators in the CNS are capable of further contributing to the alcohol-withdrawal glutamate surge, propagating a detrimental cycle of excitotoxic cellular damage in the brain (Alvarez Cooper et al., 2020; Korbecki et al., 2022). In this context, pharmacologically targeting neuroinflammatory action is possible (Boetticher et al., 2008; Roh et al., 2015) but can jeopardize peripheral immune function, specifically for those with compromised liver function from chronic alcohol use (Boetticher et al., 2008; Naveau et al., 2004). Therefore, reducing production of CXCL1 and TNFα via p38 inhibition by MW150 represents a novel approach to mitigating neuroinflammatory damage and emotional disturbance (Alvarez Cooper et al., 2020) that underlies propensity for relapse during alcohol withdrawal.

This study investigates the effects of alcohol exposure and withdrawal specifically in brain tissue divorced from the body. However, the present results of p38 inhibition in brain tissues may hold some promise for reducing peripheral damage by global MW150 treatment. CXCL1 and TNFα are considerably involved and intimately related through p38 in shaping hepatocellular response to alcohol exposure (Mandrekar & Szabo, 2009). Liver Kupffer cells become principal hubs for generating and releasing TNFα in response to alcohol (Go et al., 2018) and cellular damage (Su et al., 2018). Once released, p38 in nearby hepatocytes becomes activated and contributes to the dramatic increase in production of CXCL1 integral to produce neutrophil accumulation and hepatic tissue damage (Go et al., 2018; Su et al., 2018). Broad pharmacological inhibition (e.g., dilinoleoylphosphatidylcholine) of the p38 MAPK signaling pathway reduces TNFα expression in Kupffer cells in response to LPS administration in rats sub-chronically exposed to ethanol, although a subsequent reduction in CXCL1 was not reported (Cao et al., 2002). Therefore, it remains to be determined if precise p38α inhibition could more finely dampen peripheral inflammation as it presently has in rodent CNS tissue.

Here we found that TNFα was elevated during alcohol withdrawal only in female OHSCs. This finding is in line with previous work which reports that female OHSC tissue is more sensitive in excitatory contexts relative to male OHSC tissue (Routbort et al., 1999; Walls et al., 2013). Indeed, OHSCs possess vast sexual divergences in amino acid neurotransmitter networks as well as MAPK and calcium signaling (Weis et al., 2021). Each of these is involved in excitotoxicity and neuroinflammation and may begin to explain why male tissue was more resilient to ethanol exposure in this paradigm, although systematic investigation is warranted.

Further, while elevated levels of IL-6 by ethanol exposure was expected (Moura et al., 2022), the relative increase of IL-6 levels by MW150 treatment in BV2 cells was not. One possible explanation for why p38 inhibition increased IL-6 levels across conditions may be due to a compensatory JNK response and/or disinhibition of the NF-κB pathway, both of which can increase production of IL-6 and other cytokines in a context whereby p38 is inhibited (Jeong et al., 2013; Yang et al., 2014). Unfortunately, the experimental timeframe, acute alcohol regimen, and *in vitro* techniques provide suboptimal parameters to assess the functional ramifications of chemokine/cytokine modulation by p38 inhibition, and future studies *in vivo* would elucidate these findings.

Last, the hypothesis that MW150 could serve as a harm reduction method and/or reduce alcohol drinking behavior is premised upon the anti-inflammatory properties of p38 inhibition. P38 activation is integral in mobilizing neuroinflammation and microglial activation during alcohol withdrawal (Jung et al., 2011) which can trigger lasting alcohol “hyperkatifeia” (McNair et al., 2025) – characterized by negative affect, anxiety, general malaise, and heightened propensity for relapse due to amplification of alcohol’s negatively reinforcing properties during alcohol withdrawal (Koob et al., 2020; Koob & Vendruscolo, 2025). This phenomenon, therefore, resembles a critical and temporally-sensitive therapeutic window to both modulate neuroimmune response and reduce likelihood of hyperkatifeia, neuronal damage, and/or alcohol relapse (Koob et al., 2020; Koob & Vendruscolo, 2025; McNair et al., 2025). Given the results of the present study, p38 inhibition by MW150 may be a suitable pharmacotherapeutic mechanism to reduce “compulsive” alcohol intake during withdrawal and/or physiological harm from chronic alcohol consumption in animal models of AUD.

## 5. Conclusion

Here we provide preliminary evidence to suggest that small-molecule p38α MAPK inhibitor, MW150, may be a plausible candidate for reducing neuroinflammation during the course of alcohol withdrawal. Because p38 inhibitors are presently in clinical trials for their anti-inflammatory properties, series of follow-up studies are necessary to determine: a) the effects of this drug class with concomitant alcohol intoxication/withdrawal in an *in vivo* preclinical model; and b) the feasibility of repurposing this drug class in clinical trials for those with chronically-relapsing AUD.
